# Supplementary material for: A Retro-Aldol Reaction Prompted the Evolvability of a Phosphotransferase System for the Utilization of a Rare Sugar
Source: Microbiol Spectr. 2023 Feb 14;11(2):e03660-22. doi: 10.1128/spectrum.03660-22 (PMC10101011; doi:10.1128/spectrum.03660-22)
Supplement: Supplemental file 1 — Supplemental material. Download spectrum.03660-22-s0001.pdf, PDF file, 5.1 MB [file spectrum.03660-22-s0001.pdf]

Supplementary information

A retro-aldol reaction prompted the evolvability of a phosphotransferase system for the utilization of a rare sugar

Yunhye Joo[a,¶], Jae-Yoon Sung[a,¶], Sun-Mi Shin[b,£], Sun Jun Park[c,d], Kyoung Su Kim[a], Ki Duk Park[c,d], Seong-Bo Kim[e], and Dong-Woo Lee [a,*]

[a]*Department of Biotechnology, Yonsei University, Seoul 03722, Republic of Korea.*

[b]*School of Applied Biosciences, Kyungpook National University, Daegu 41566, Republic of Korea.*

[c]*Convergence Research Center for Brain Science, Korea Institute of Science & Technology (KIST), Seoul 02792, Republic of Korea.*

[d]*Division of Bio-Medical Science & Technology, KIST School, Korea University of Science and Technology, Seoul 02792, Republic of Korea.*

[e]*Department of Bio-Living Engineering, Yonsei University, Seoul 03722, Republic of Korea.*

Running title: Evolution of bacteria toward a non-utilizable sugar

*To whom correspondence should be addressed: **Dong-Woo Lee**

Department of Biotechnology, Yonsei University, Seoul 03722, South Korea.

Tel.: +82-2-2123-2886; Fax: +82-2-362-7265; E-mail: leehicam@yonsei.ac.kr

[¶]These authors contributed equally to this work.

[£]Current address: *Research Institute of Biotechnology, CJ CheilJedang Corp., CJ Blossom Park. Suwon 16495, South Korea.*

Supplementary Table 1. Whole genome sequencing of the YF1^{BL_GatY}, and YF2 strains

Position	Mutation	Reference nucleotide	Altered nucleotide	Gene	Location	Strain
749,894.. 749,895	SUB	AA	GT		*lacUV5* promoter	YF1^{BL_GatY}, YF2
749,902	SUB	G	A			
937,377	SNP	C	T		Upstream of *trxB* and *lrp*	YF1^{BL_GatY}, YF2
1,100,143.. 1,100,144	INS	-	15386..16725 (1,340 bp)	*Ins*	tRNA-Ser coding gene	YF1^{BL_GatY}, YF2
2,157,100	SNP	C	A	*fruK*	FruK coding gene	YF1^{BL_GatY}, YF2
2158366.. 2158432	DEL	TGAAACGATTCAG CCTCTATGAGAAA AAAAGCGCCAAC CTGGCTTAGGGTT AAAGACAAGATC GCGC (67 bp)	-		Cra binding site (5'-UTR of *fru* operon)	YF1^{BL_GatY}, YF2
3,144,807.. 3,144,823	DEL	GGTGCCTGTCACT CGCT (17 bp)	-	*agaR*	AgaR coding gene	YF2

SUB, substitution; SNP, single nucleotide polymorphism; INS, insertion. Insertion sequence and genetic features in 1,100,143..1,100,14 (tRNA-Ser) positions; IS3-like element IS150 family transposase (1,340 bp)

Supplementary Table 2. Strains and plasmids used in this study

Strains or plasmid	Information	Genotype, relevant characteristics, or sequence	Designation
Strains	Harboring plasmids		
E. coli DH5α		F– Φ80lacZΔM15 Δ(*lacZYA-argF*) U169 *recA*1 *endA*1 *hsdR*17 (rK–, mK+) *phoA supE*44 λ– thi-1 *gyrA*96 *relA*1	
E. coli BL21(DE3)		F– *ompT gal dcm lon hsdSB*(*rB- mB-*) λ(DE3 [*lacI lacUV5*-T7 gene 1 *ind*1 *sam*7 *nin*5]	
Bacillus licheniformis		ATCC 14580	
E. coli BL21(DE3)	pET28a-BL_GatY	WT	
	pET28a-BL_GatY	Supporting Table 1.	$YF1^{BL\_GatY}$
		Plasmid curing	YF1
		Supporting Table 1.	YF2
		CraBS_Δ67bp	sC
		*fruK*_115G>T (FruK_A39S)	sF
		Δ*agaR*	sA
	pET28a-BL_GatY	CraBS_Δ67bp	sC^{BL_GatY}
	pET28a-BL_GatY	*fruK*_115G>T (FruK_A39S)	sF^{BL_GatY}
		CraBS_Δ67bp, *fruK*_115G>T (FruK_A39S)	dCF
		CraBS_Δ67bp, Δ*agaR*	dCA
		*fruK*_c.115G>T (FruK_A39S) Δ*agaR*	dFA
	pET28a-BL_GatY	CraBS_Δ67bp, *fruK*_115G>T (FruK_A39S)	dCF^{BL_GatY}
		CraBS_Δ67bp, *fruK*_115G>T (FruK_A39S), Δ*agaR*	tCFA
		CraBS_Δ67bp, *fruK*_115G>T (FruK_A39S), $AgaR^{PTC}$	$tCFA^{PTC}$
	pET28a-BL_GatY<br>PET22b-EC_AraA	Δ*araA*::FRT	$YF1^{BL\_GatY,\ EC\_AraA}$
	pET28a-BL_GatY<br>PET22b-EC_AraA<br>Mutant library	Δ*araA*::FRT	$YF1^{BL\_GatY}$, $^{EC\_AraA\ ML}$
E. coli YF2		Δ*fruA*::FRT	YF2 Δ*fruA*
		Δ*gatABC*::FRT	YF2 Δ*gatABC*
		Δ*fruK*::FRT	YF2 Δ*fruK*
		Δ*kbaY*::FRT	YF2 Δ*kbaY*
Plasmids			Sources

pET15b		
pET22b(+)		
pET28a(+)		
pET22b(+)-EC_AraA		In this study
pET28a(+)-BL_GraY		In this study
pRedET	λ phage red γ,β,α-producing vector, pBAD-promoter; ori101 *Tet*^{r}	Gene bridges
FRT-PGK-gb2-neo-FRT template DNA	FRT flanked *Kan*^{R} cassette	Gene bridges
707-FLP	Flp recombinase-producing vector; pSC101 ori cI578 *Tet*^{r}	Gene bridges
pCas9	*repA101*(Ts) *kan* P_{cas}-*cas*9 P_{araB}-*Red lacI*^{q} P_{trc}-sgRNA-*pMB1*	(Jiang et al. 2015)
pTargetF-*cat*	*pMB1 aadA* sgRNA-cat	(Jiang et al. 2015)

Mother strain genotype of *E. coli* BL21(DE3) for the ALE experiment: F– *ompT gal dcm lon hsdSB*(*rB- mB-*) λ(DE3 [*lacI lacUV5*-T7 gene 1 *ind*1 *sam*7 *nin*5]

Supplementary Table 3. Primers used for the cloning process

Strain	Genotype, relevant characteristics, or sequence
Primers	
araA-FKF_F	5′-CGCCCCGGCACAGGCTGCCCAGGCCGTTGCGACTCTATAAGGACACGATAA-ATTAACCCTCACTAAAGGGCGG-3′
araA-FKF_R	5′-GATAAGACGCGTCAGCGTCGCATCAGGCGTTACACACCTGATGCGACTACT-AATACGACTCACTATAGGGCTC-3′
araA-con-A	5′-ACAACGCTTTGAACAGCTTTATCGC-3′
araA-con-B	5′-CCTGGCGTTTGAGATCTTCTAACAT-3′
EC_*ara*A *Nde*I_F1	5′-CATATGACGATTTTTGATAATTATGAAGTGTGG-3′
EC_*ara*A *Hind*III_R	5′-AAGCTTTTAGCGACGAAACCCGTAATAC-3′
EC_*ara*A *Nde*I_F2	5′-CGCCATATGACGATTTTTGATAATTATGAAG-3′
EC_*ara*A *Xho*I_R	5′-ATTTCTCGAGTTAGCGACGAAACCC-3′
*BL_gatY Nde*I_F	5′-ATCAATTGATAGCATATGTACGTGGTATCGACAAAGCAGATGC-3′
*BL_gatY Xho*I_R	5′-ATCAATTGATAGCTCGAGAATTATGCCCTGCCCTCGCAACCAC-3′
*EC_gatY Nde*I_F	5′-ATCAATTGATAGCATATGCTGACAAATACGAAAAAAATGCTGCTG-3′
*EC_gatY Xho*I _R	5′-ATCAATTGATAGCTCGAGAATTAGTATCTGTCATTGCTCATGCACATC-3′
*EC_kbaY Nde*I_F	5′-ATCAATTGATAGCATATGAGCATTATCTCCACTAAATATCTGTTACAG-3′
*EC_kbaY Xho*I _R	5′-ATCAATTGATAGCTCGAGAATTATGCTGAAATTCGATTCGCTGAACC-3′
*EC_ fbaA Nde*I_F	5′-ATCAATTGATAGCATATGTCTAAGATTTTTGATTTCGTAAAACCTGG-3′
*EC_ fbaA Xho*I _R	5′-ATCAATTGATAGCTCGAGAATTACAGAACGTCGATCGCGTTCAG-3′
EC_*fru*K_WT *Nde*I_F	5′-GATAGTAGCAACACGTCTGCTCATATGCTATCAATTGAT-3′
EC_*fru*K_WT *Xho*I _R	5′-GCGTCGACTTACAACCTTTTAACTGATTCTCGAGCTATCAATTGAT-3′
*fru*A-FKF_F	5′- GATGGCGCGCGTCGACTTACAACCTTTTAACTGACAGCAGGAGAGGCATAAATTAACCCTCA CTAAAGGGCG -3′
*fru*A-FKF_R	5′- TCATCCAATGATACAGGGCAGGAAATTTCTGCCCTGTAACACACCTTTTATAATACGACTCAC TATAGGGCTC-3′
*fru*K-FKF_F	5′- GTGTTGCTACTATCACCCTTAATCCGGCTTATGACCTTGTTGGTTTCTGCAATTAACCCTCACT AAAGGGCG-3′
*fru*K-FKF_R	5′- CTGCTGTCAGTTAAAAGGTTGTAAGTCGACGCGCGCCATCATTGCGGCCATAATACGACTCA CTATAGGGCTC-3′

*kba*Y-FKF_F	5′-TGGTGCAAGGCGTAATCATTCATCCGTGGCAGGCATAAGAGGATCGCATTAATTAACCCTCACTAAAGGGCG-3′
*kba*Y-FKF_R	5′-CCCGATATTAATCGGGAAGTACATTGGGATAAATCATAAAAAACTAATGATAATACGACTCACTATAGGGCTC-3′

[a]Restriction enzyme sites are underlined.

Supplementary Table 4. Primers used for the qRT-PCR experiment.

Primers	Sequence
*fruB*_F	5′-TTGTGGTGCGCAATGAACAC-3′
*fruB*_ R	5′-TTAACGCCAAGTGCCACAAC-3′
*fruK*_ F	5′-ACAAGGTATCGCGCATGTTG-3′
*fruK*_ R	5′-TGGATTCACGCATCAGCAAG-3′
*fbaA*_ F	5′-AAACCTGGCGTAATCACTGG-3′
*fbaA*_ R	5′-CCGTTGGAGAACTGAACGAT-3′
*fbaB*_ F	5′-TGTGGGCCTATTTGCGTAAC-3′
*fbaB*_ R	5′-ATAGCCGCCGTTATTTTCCG-3′
*ECgatY*_ F	5′-TGGTGGATTTTTGCCATCGC-3′
*ECgatY*_ R	5′-ACAACGCATCGGCTTCATTG-3′
*gatZ*_ F	5′-TTGCTTTGCTGCGGAAAGTG-3′
*gatZ*_ R	5′-TGCGTGCGTAAAGTATTGGC-3′
*gatA*_ F	5′-TGCAATACCGCATTGTGAGG-3′
*gatA*_ R	5′-ATTGCTGCTGCGGATTTTCC-3′
*gatB*_ F	5′-AAGGCATGCCGTGAACTAAC-3′
*gatB*_ R	5′-GGCGGCGGAAGAAATTAAAGAG-3′
*gatC*_ F	5′-AGCGATGGCGGAAAATTTCG-3′
*gatC*_ R	5′-AGAATCGCAATCGGAATCGC-3′
*pfkA*_ F	5′-TGGCTGTGAATTCGTTGTGG-3′
*pfkA*_ R	5′-AAATGCGCCAGTTCGTCAAC-3′
*pfkB*_ F	5′-AGGCGCGGCATTAAATGAAG-3′
*pfkB*_ R	5′-AGCGGATCCCTTGTTTTTGC-3′
*manX*_ F	5′-AACGTTAATGGCCCGTGATG-3′
*manX*_ R	5′-TTTGGCCCCATTGGTTTTGC-3′
*manY*_ F	5′-ACATGCTGAATGCGATTCCG-3′
*manY*_ R	5′-TTGGTGAATGCTGCGGTTAC-3′
*ptsH*_ F	5′-TGACTTCCAACGGCAAAAGC-3′
*ptsH*_ R	5′-TGTTCAACCGCTTTCTGCTC-3′
*ptsI*_ F	5′-TAAAATGCGCGCTGTTCAGG-3′
*ptsI*_ R	5′-TTCAACGTCACGAACCGTAC-3′
*ptsG*_ F	5′-TTGCCGGTAAACGCTTTGTG-3′
*ptsG*_ R	5′-AGGCAACGTTCGATGAAACC-3′
*T7RNAP*_ F	5′- -ACGCTTCCGCAAGATGTTTG-3′
*T7RNAP*_ R	5′-AGTCGTTGATGCGTGCAATC-3′
*Cra*_ F	5′-ATTGTTTCGACGTCGTTGCC-3′
*Cra*_ R	5′-AACTCTTCCGCCAGCATTTC-3′
*fruA*_ F	5′-GCCATGCGAAACCTTACACT-3′
*fruA*_ R	5′-CAGAACCACGGGTTTCAACT-3′
*kbaY*_ F	5′-CCTCGAAGTGTGCAGTGAAA-3′
*kbaY*_ R	5’-GCGAATATCATCCAGCGATT-3’
*kbaZ*_ F	5’-GAAAGCCTGTTCTGGTCTGC-3’
*kbaZ*_ R	5’-AGCGAAAGCGTCATCAATCT-3’
*manZ*_ F	5’-ATATTCGTGGCGTCTTCCTG-3’
*manZ*_ R	5’-GAACGGCTGGGTGTTAAAGA-3’

*mak_* F	5'-GCTAACTGTCTGGCGGTTTC-3'
*mak_* R	5'-GATTGTGTCCCCACTCACCT-3'
*mtlA_* F	5'-TTCCCGTATGTGCTGATGAA-3'
*mtlA_* R	5'-GCGAAGTAAGCACCTTTTGG-3'
*crr_* F	5'-GTTTCCGACGACAAGAAGGA-3'
*crr_* R	5'-GCGTGGTTGGTTTCAAAGAT-3'
*galP_* F	5'-AACATGATCGTTGGCGCAAC-3'
*galP_* R	5'-TTCCAGCGAAACGTGTTTGG-3'
*araA_* F	5'-TTGCCGCACAGATCAAGTTC-3'
*araA_* R	5'-TTTGTGTTGCAGGCGTCATG-3'
*BLgatY_* F	5'-TCAAAAACGGGTTTCAGTCC-3'
*BLgatY_* R	5'-CGGGGGCCATATATTTTCTT-3'
*tolQ_* F	5'-TATCGCAGAAGCGTTGATTG-3'
*tolQ_* R	5'-CCCTTGTTGCTCTCGCTAAC-3'
*rpoD_* F	5'-CAATGCTCCGTTGCTGAATA-3'
*rpoD_* R	5'-TCTTCCTGGGAAAGCTCAGA-3'
*gapA_* F	5'-AGGTCTGATGACCACCGTTC-3'
*gapA_*R	5'-GGAACGCCATACCAGTCAGT-3'
*agaR_*F	5'-TCGCAATTACGTCAGTGTGC-3'
*agaR_*R	5'-ACTGAACACCGCGATGAAAC-3'

Supplementary Table 5. Kinetic parameters of ECAI (wild-type) and L-AI variants

Enzyme	K_M (mM)	V_{max} (U/mg)	k_{cat} (min^{-1})[a]	k_{cat}/K_M $(\mathrm{mM}^{-1}\mathrm{min}^{-1})$
L-Arabinose				
Wild-type	86.2 ± 13.0 [b]	35.3 ± 2.5	1979.4	23.0
H17R/R159S/V168A	164.7 ± 34.4	32.8 ± 4.1	1840.5	11.2
E22D/M95L/H157L	135.5 ± 11.7	34.9 ± 1.4	1954.6	15.7
V368A/E493D	87.5 ± 14.4	31.8 ± 2.5	1783.9	20.4
D-Galactose				
Wild-type	1317 ± 481	1.4 ± 0.4	80.0	0.06
H17R/R159S/V168A	512 ± 46	1.1 ± 0.1	60.6	0.12
E22D/M95L/H157L	830 ± 267	0.8 ± 0.2	47.3	0.06
V368A/E493D	681 ± 60	0.9 ± 0.1	51.7	0.08

Kinetic parameters were obtained by fitting the experimental data to the Michaelis–Menten equation, as follows: $V = V_{max}[S]/(K_M+[S])$. All data were obtained in triplicate. [a] k_{cat} is the number of substrate molecules reacted per active site per min. [b] Data are mean ± standard deviation.

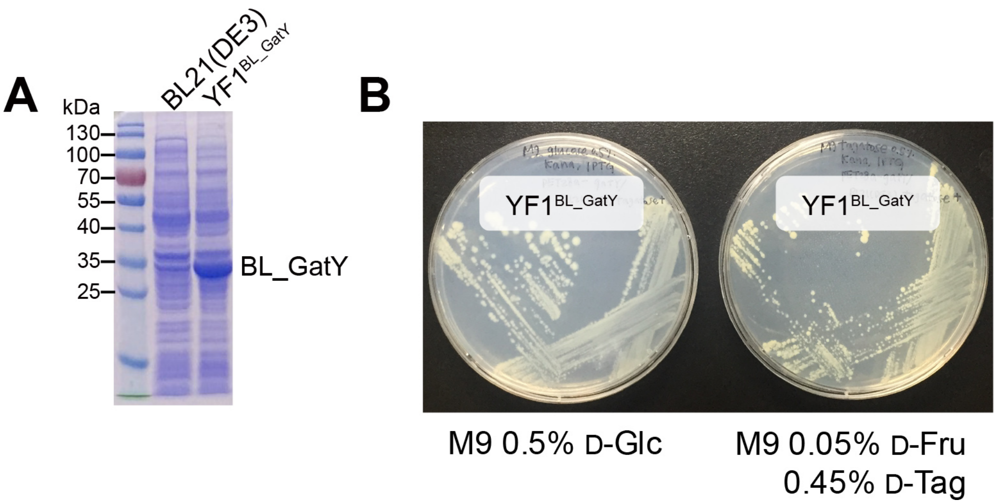

Supplementary Figure 1. Analysis of recombinant gene expression and the phenotype of the $\mathbf{YF1^{BL\_GatY}}$ strain in M9 sugar solid media. A. SDS-PAGE of whole-cell lysates of *E. coli* BL21(DE3) cultured in LB media, and $YF1^{BL\_GatY}$ cultured in M9 media supplemented with 0.05% D-Fru and 0.45% D-Tag. **B.** *E. coli* $YF1^{BL\_GatY}$ strains in M9 solid media supplemented with 0.5% D-Glc or supplemented with M9 with 0.05% D-Fru and 0.45% D-Tag.

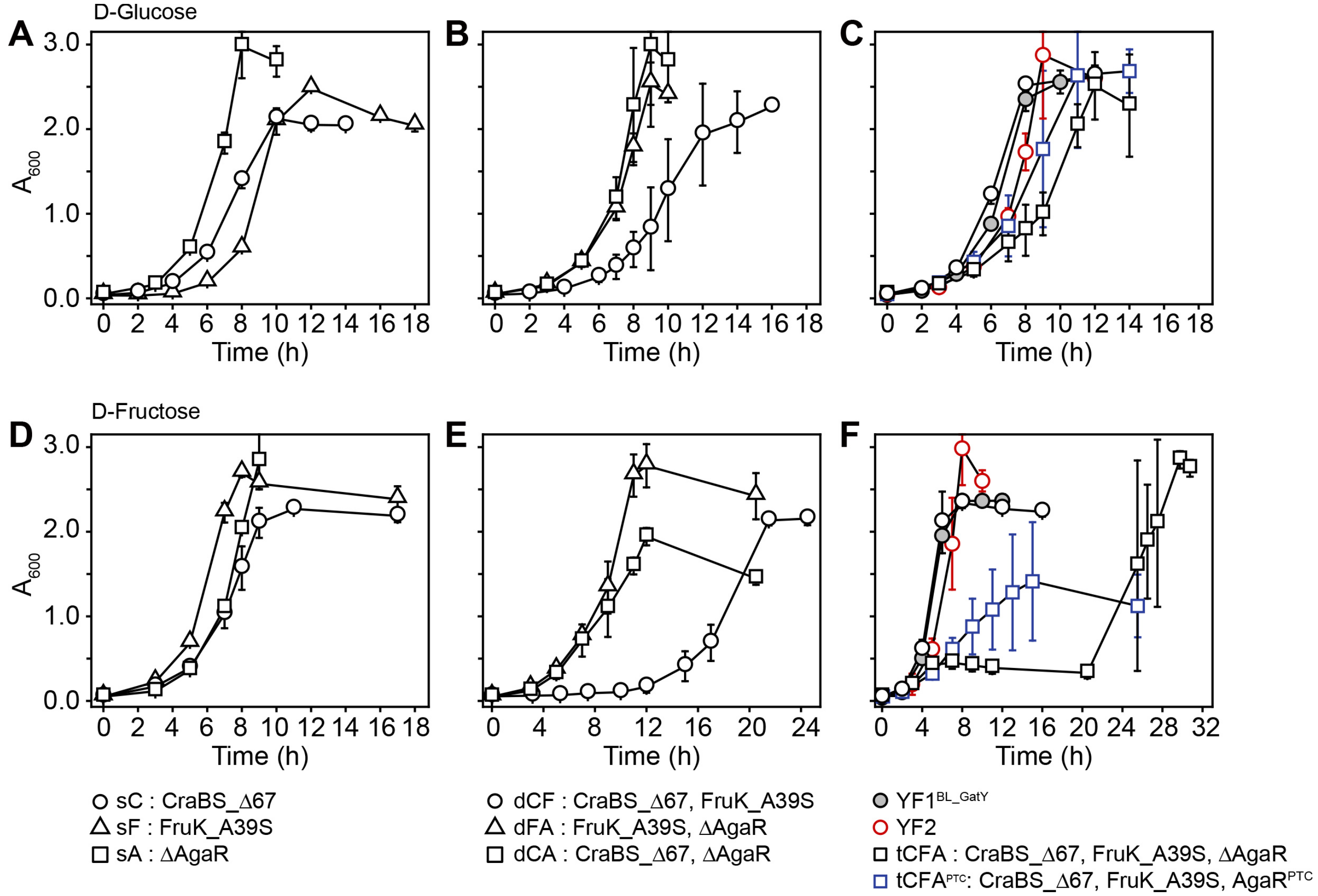

Supplementary Figure 2. Growth profile of three types of mutant strains in M9 sugar media. A. Single mutant strains, sC, CraBS_Δ67; sF, FruK_A39S; sA, Δ*agaR*. **B.** Double mutant strains, dCF, CraBS_Δ67, FruK_A39S; dFA, FruK_A39S, Δ*agaR*. **C.** Triple mutant strains, tCFA, CraBS_Δ67, FruK_A39S, Δ*agaR*; tCFA^{PTC}, CraBS_Δ67, FruK_A39S, AgaR^{PTC} in M9 media supplemented with 0.5% D-Glc**. D.** Single mutant strains, **E.** double mutant strains, **F.** triple mutant strains, in M9 media supplemented with 0.5% D-Fru**.**

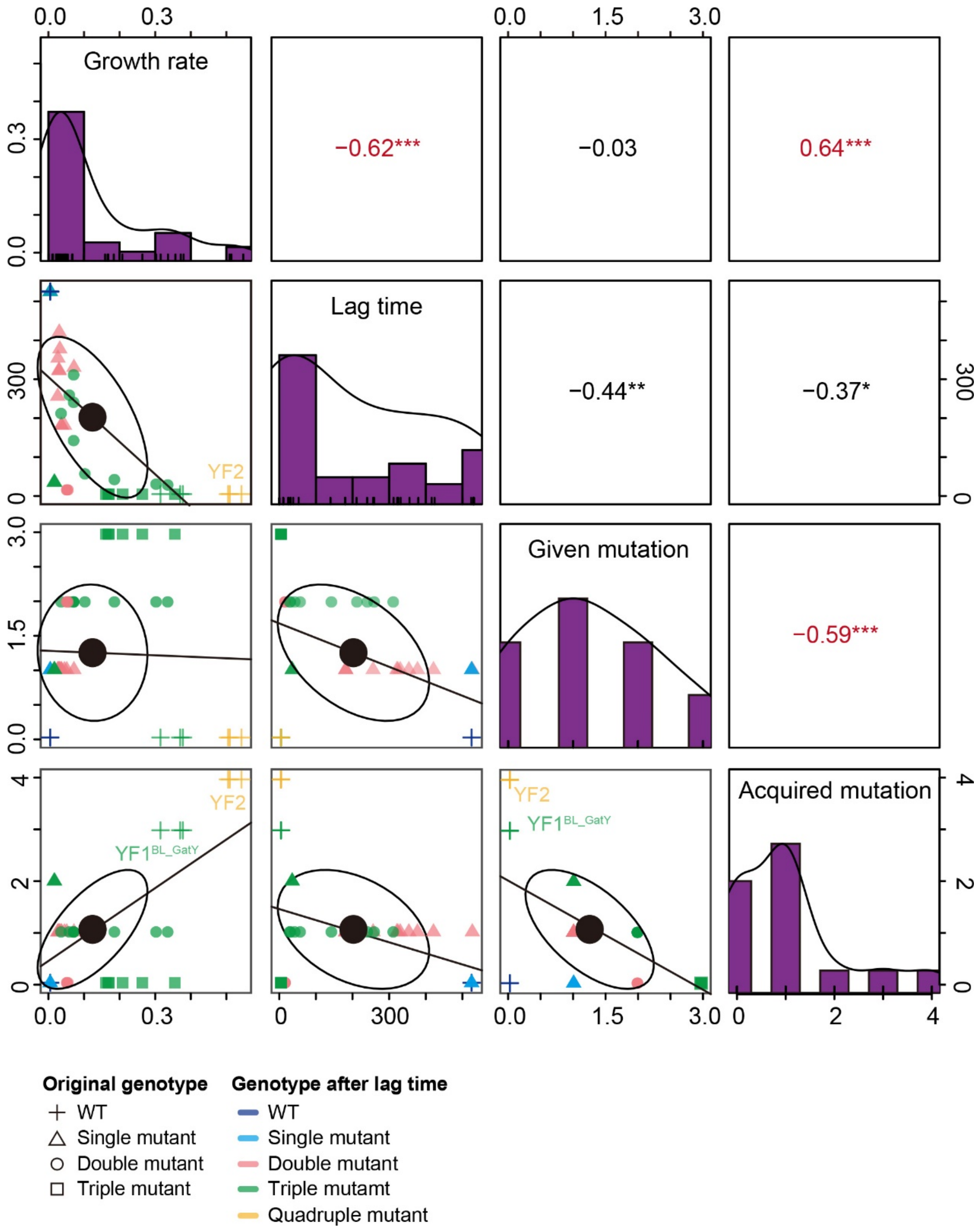

Supplementary Figure 3. Correlation analysis of ALE-induced mutant strains derived from M9 media supplemented with 0.05% D-Fru and 0.45% D-Tag. Pearson's correlation coefficients between four numeric variables, growth rate, lag time to obtain D-Tag utilization, number of given mutations by the cas9 system, and number of acquired mutations from evolution with a lag period. WT indicates the original genotype (i.e., the genotype before culture in D-Tag); cross; single mutant, triangle; double mutant, circle; triple mutant, square. Changed genotype, obtained after culture in D-Tag requiring a lag period, WT, blue line; single mutant, sky blue line; double mutant, red line; triple mutant, green line; quadruple, yellow line.

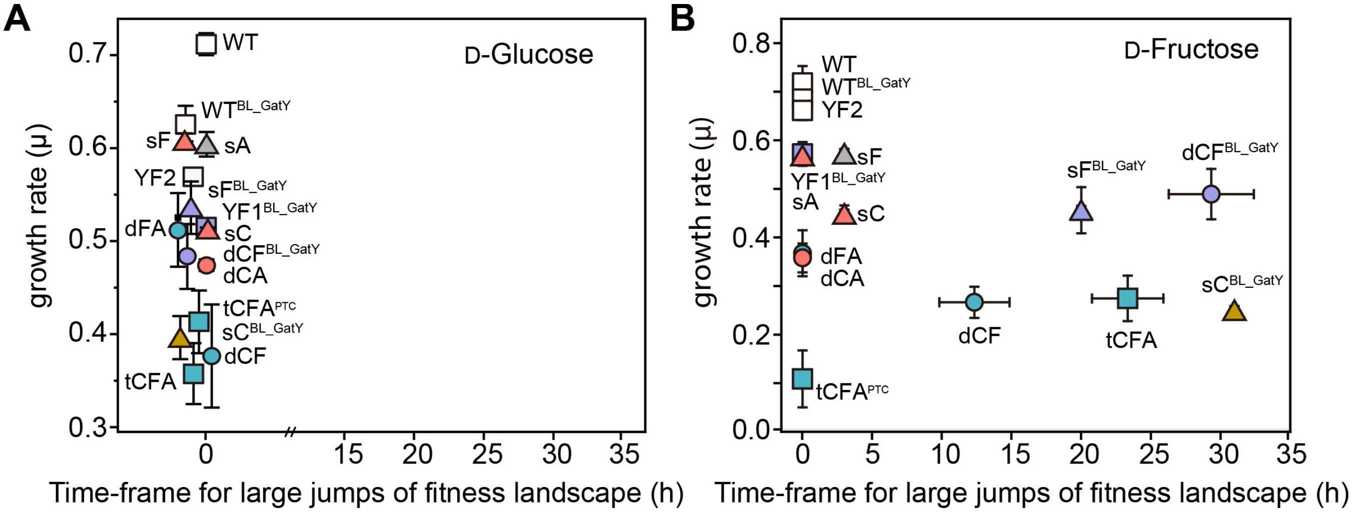

Supplementary Figure 4. Effects of single, double, triple mutations on growth phenotypes in M9 sugar media. A. Growth rates of mutant strains in M9 media supplemented with 0.5 % D-Glc. **B.** Comparison of mutant strains with different growth rates and lag periods in M9 media supplemented with 0.5% D-Fru.

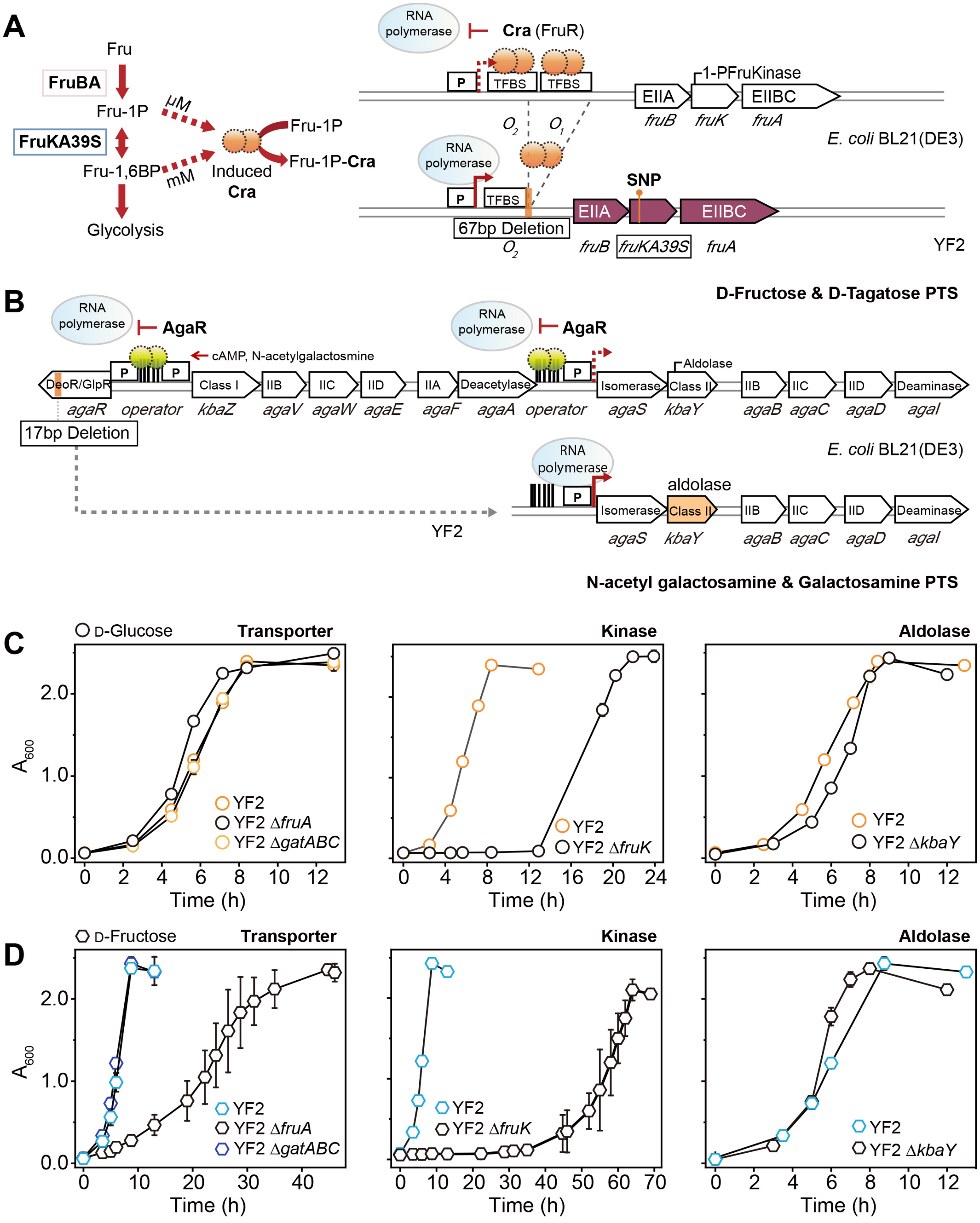

Supplementary Figure 5. Identification of a novel D-Tag catabolic pathway in *E. coli*. A. Prediction of the effects of the deletion of the CraBS region on *fru* operon expression and **B.** the deletion of the *agaR* on *kbaY* gene expression, encoding D-Tag-1,6-BP aldolase. **C.** Phenotypic change of mutant strains with the deletion of transporter, kinase, aldolase coding genes in M9 media supplemented with 0.5% D-Glc. **D.** Phenotypic change of mutant strains with the deletion of transporter, kinase, or aldolase genes in M9 media supplemented with 0.5% D-Fru.

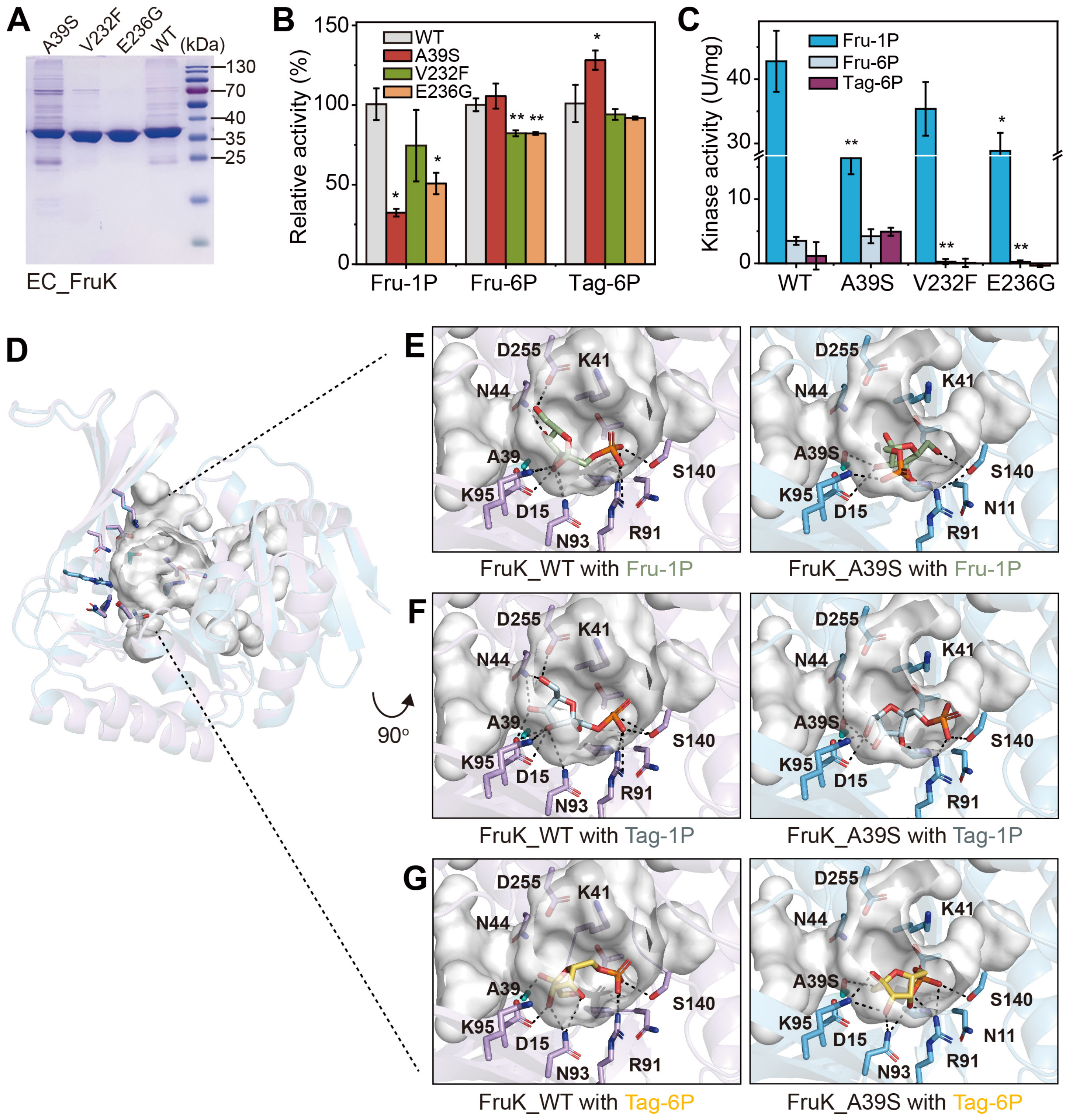

Supplementary Figure 6. Activity assay of 1-phosphofructose kinase variants as a phosphorylated tagatose kinase. A. SDS-PAGE of purified kinases, WT, and three 1-phosphofructose kinase variants. The overexpressed enzyme was analyzed by 12% SDS-PAGE (kDa, molecular weight marker) **B**. Relative activity (%) of FruK and **C.** specific activity (U/mg) of FruK with three types of phosphorylated ketohexose. **D.** 3D-structural modeling of FruK WT and A39S variant with **E.** D-Fru-1P substrate, **F.** D-Tag-1P, and **G.** D-Tag-6P.

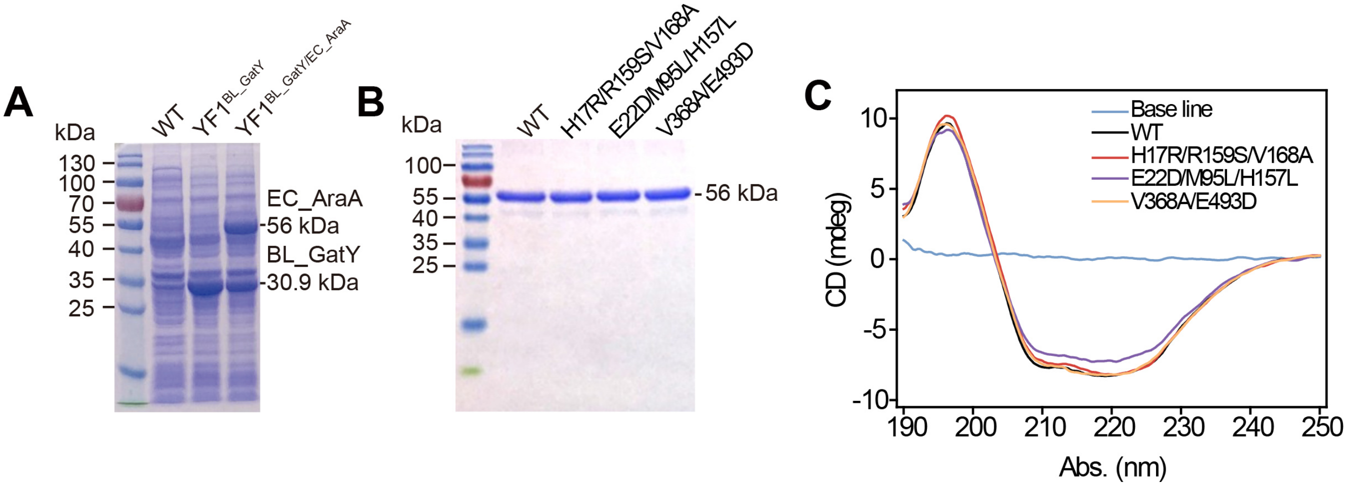

Supplementary Figure 7. Analysis of recombinant gene expression and the influence of mutations on the structural integrity and activity of EC_AraA and its variants. A. SDS-PAGE analysis of recombinant *gatY* and *araA* genes. The overexpressed enzyme was analyzed by 12% SDS-PAGE (WT, whole-cell lysates of Δ*araA E. coli* BL21 (DE3); YF1^{BL_GatY}, whole-cell lysates of Δ*araA* YF1 strain harboring pET-28a(+)-BL_GatY; YF1$^{BL_GatY/EC_AraA}$, whole-cell lysates of Δ*araA* YF1 strain harboring pET-28a(+)-*BL_gatY* and pET-22b(+)-*EC_araA*. **B.** SDS–PAGE analysis of purified EC_AraA WT and its variants: EC_AraA_WT, EC_AraA_H17R/R159S/V168A, EC_AraA_E22D/M95L/H157L, EC_AraA_V368A/E493D. **C.** The CD spectra of EC_AraA WT and its mutant AraAs.

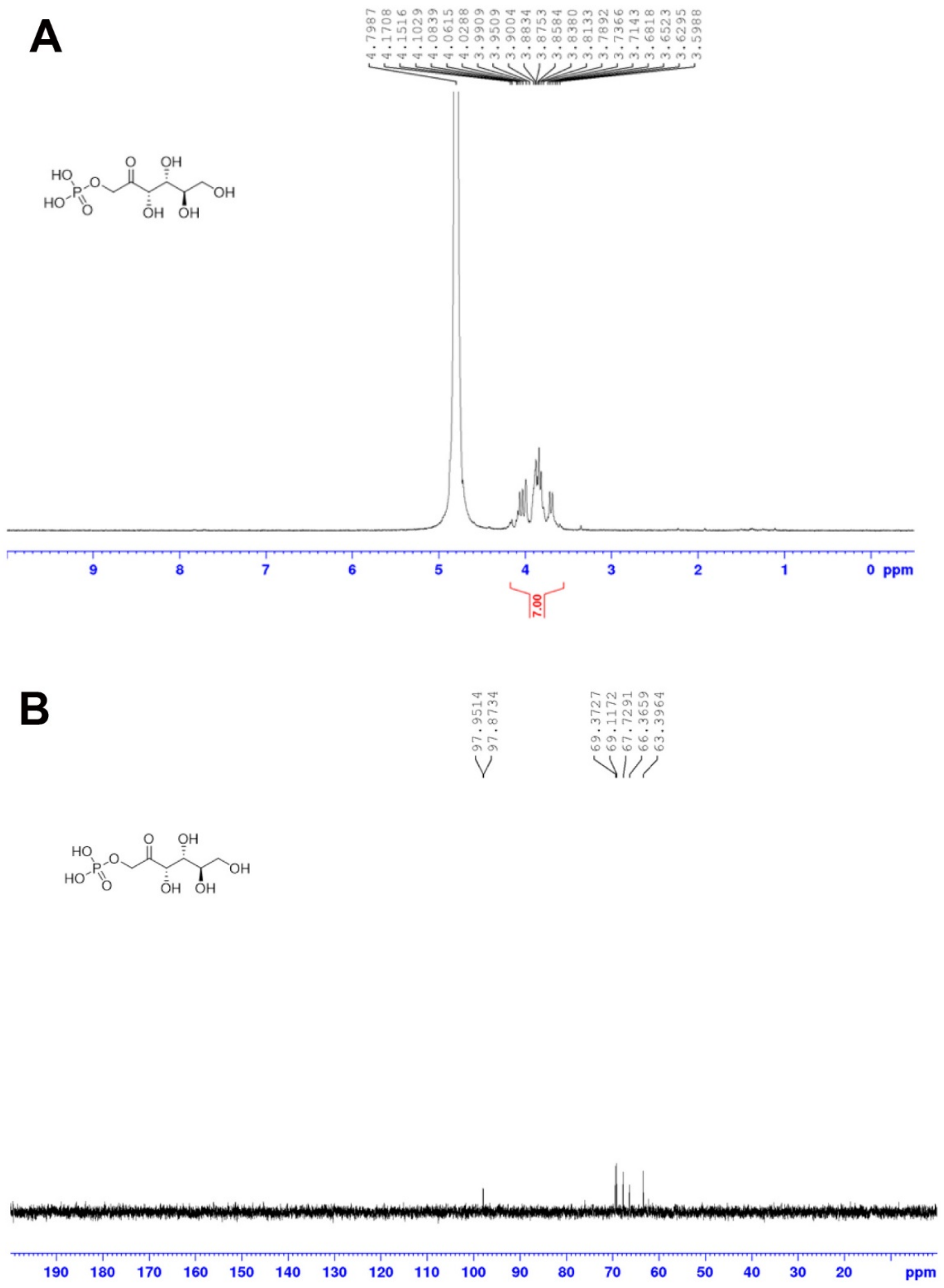

Supplementary Figure 8. ¹H and ¹³C-NMR spectra of fructose-1-phosphate. A. The ¹H NMR spectrum was recorded at 400 MHz using a Bruker spectrometers. **B.** The ¹³C NMR spectrum was recorded at 100 MHz using a Bruker spectrometer. NMR data were analyzed with Bruker Topspin 3.6.2.
